# Identification of the immune subtype of ovarian cancer patients by integrated analyses of transcriptome and single-cell sequencing data

**DOI:** 10.1038/s41598-022-17645-7

**Published:** 2022-08-02

**Authors:** Sixue Wang, Xi Wang, Xiaomeng Xia, Tingting Zhang, Mingyu Yi, Zeying Li, Li Jiang, Yalan Yang, Jie Fu, Xiaoling Fang

**Affiliations:** 1 Department of Obstetrics and Gynecology, The Second Xiangya Hospital of Central South University, Changsha, China; 2 Department of General Surgery, The Second Xiangya Hospital of Central South University, Changsha, China

**Keywords:** Cancer, Cancer microenvironment, Gynaecological cancer

## Abstract

Ovarian cancer (OC) is one the most life-threatening cancers affecting women’s health worldwide. Immunotherapy has become a promising treatment for a variety of cancers, but the therapeutic effects in OC remain limited. In this study, we constructed a macrophage risk score (MRS) based on M1 and M2 macrophages and a gene risk score (GRS) based on the prognostic genes associated with MRS. Next, cell–cell communication analysis was performed using single-cell RNA (scRNA) sequencing data. Survival status and immune characteristics were compared between the high- and low-score groups separated by MRS or GRS. Our results suggested that MRS and GRS can identify the immune subtypes of OC patients with better overall survival (OS) and inflammatory immune microenvironment. Moreover, M1 and M2 macrophages may affect the prognosis of OC patients through signal communication with CD8 T cells. Finally, functional differences between the two groups separated by GRS were elucidated. Taken together, this study constructed two useful models for the identification of immune subtypes in OC, which has a better prognosis and may have a sensitive response to immune checkpoint inhibitors (ICIs). The hub genes for the construction of GRS may be potential synergetic targets for immunotherapy in OC patients.

## Introduction

Ovarian cancer (OC) is one the most life-threatening cancers affecting women’s health worldwide^1,2^. Many patients suffering from OC lose the opportunity for optimal surgery because of occult onset and no obvious early symptoms^3,4^. Moreover, benefits of platinum-based chemotherapy and maintenance therapy for patients with advanced OC patients remain unsatisfactory^5–7^. Therefore, it is urgent to clarify the mechanisms of ovarian carcinogenesis to find more sensitive diagnostic markers, as well as more effective therapeutic targets or synergetic targets combined with classical therapeutic methods.

Immunotherapy has become a promising therapeutic method in recent years^8–10^. Monotherapy with immune checkpoint inhibitors (ICIs) or chimeric antigen receptor-T (CAR-T) cells has shown good efficacy in hematological tumors and non-small-cell lung cancer (NSCLC)^11–13^. In addition, immunotherapeutic drugs combined with other classical drugs have also shown good efficacy in a variety of tumors, such as ICIs combined with angiogenesis inhibitors for liver cancer or combined with BRAF inhibitors for advanced melanoma with BRAF V600 mutation^14,15^. Moreover, the proportion of M1/M2 macrophages has also been preliminarily studied in the prognosis evaluation of ovarian cancer^16,17^. At present, there are also some studies related to immunotherapy for OC, such as programmed cell death-ligand 1 (PD-L1) or programmed cell death-1 (PD-1) combined chemotherapy or angiogenesis inhibitors for recurrent OC, which are in different stages of clinical trials^18–21^. However, the response of OC to immunotherapy is still very limited. Under these conditions, it is urgent to elucidate the immune characteristics of OC patients for the identification of immune subtypes and to search for synergetic targets of immunotherapy.

In this study, immune characteristics were systematically investigated in OC patients, and risk scores based on M1 and M2 macrophages (macrophage risk score, MRS) or significant MRS-related prognostic genes (gene risk score, GRS) were subsequently constructed. Both the MRS and GRS could identify the immune subtype of OC patients who had a better prognosis and an inflammatory immune microenvironment. Moreover, M1 and M2 macrophages may play roles in OC by affecting the function of CD8 T cells. Taken together, this study constructed a useful model for the identification of immune subtypes in OC patients who may be sensitive to immunotherapy, and potential synergetic targets for the immunotherapy of OC patients were preliminarily identified.

## Methods

### Data acquisition and processing

Bulk-sequencing data in fragments per kilobase million (FPKM) or count forms of ovarian serous cystadenocarcinoma from TCGA were acquired from UCSC-Xena (https://xena.ucsc.edu/), while the corresponding clinicopathological information were acquired from cBioPortal (https://www.cbioportal.org/). FPKM data were transformed into transcripts per million (TPM) data before analysis. After processing, a total of 322 patients with complete clinical data and overall survival (OS) ≥ 1 month were enrolled in this study as a training cohort. Clinicopathological characteristics were each divided into two groups: age (≥ 60 years, < 60 years), stage (stages I–II, III–IV), histological grade (low-grade: G1 and G2, high-grade: other grades), longest dimension (≥ 1 cm, < 1 cm), tumor site (unilateral, bilateral) and race (white, nonwhite). Next, microarray and survival data of OC patients with OS ≥ 1 month from GSE53963 (n = 170), GSE9891 (n = 276), GSE13876 (n = 157), GSE26712 (n = 184), GSE49997 (n = 194) and GSE140082 (n = 376) were downloaded from the GEO database (https://www.ncbi.nlm.nih.gov/geo/) as validation cohorts^22–26^. All methods were carried out in accordance with the Declaration of Helsinki guidelines and approved by the Clinical Research Ethics Committee of the Second Xiangya Hospital, Central South University. All data analyzed in this study were downloaded from public databases, and the informed consent have been obtained by the data provider.

### Abundance estimation and prognosis prediction of immune cells

The abundances of 22 immune cell types in each OC sample were calculated by CIBERSORT in R software^27^. Subsequently, univariate and multivariate Cox analyses were conducted to identify the immune cells that were significantly associated with prognosis. A risk score (MRS) was constructed based on the most prognostic immune cells (M1 macrophages and M2 macrophages) and their corresponding risk coefficients. Next, the prognostic value of the risk score was evaluated by risk plots and Kaplan–Meier plotter curves. Finally, the immune landscape discrimination value of the risk score was evaluated by gene set variation analysis (GSVA), and the expression levels of the major immune checkpoints (CTLA-4, PDCD-1 and CD274) and 22 immune cell types between the different groups separated by the optimal cutoff value of the risk score according to the “roc” method.

### Identification and prognostic analyses of the hub genes associated with immune cells

The gene expression modules and their associations with the 22 immune cell types in OC were identified by WGCNA^28^. The genes in the modules closely associated with M1 macrophages or M2 macrophages were selected for further analyses. Seven genes were screened out by least absolute shrinkage and selection operator (LASSO) analysis, and the prognostic values of these genes were further analyzed by univariate and multivariate Cox analyses. Subsequently, a risk score (GRS) was constructed based on the remaining prognostic genes (P < 0.05) and their corresponding risk coefficients. The prognostic value and the immune landscape discrimination value of the GRS were evaluated consistent with the methods of MRS. Finally, the potential therapeutic drugs targeting these significant prognostic genes were screened by CellMiner, a web-based tool for exploring transcript and drug patterns in the NCI-60 cell line set.

### Acquisition and processing of scRNA sequencing data

ScRNA sequencing data of four primary OC tumors, two peritoneal metastases and two relapsed tumors were downloaded from the GEO database (GSE130000). Data processing and analysis were performed using the “Seurat” package^29^. After filtering by the criteria of min.cells = 3 and min.features = 200, a total of 32,078 cells (including 13,366 primary tumor cells, 5385 peritoneal metastasis tumor cells and 13,327 relapse tumor cells) remained for further analysis. Next, data normalization and screening of the 3000 highly variable genes (HVGs) were conducted by the “SCTransform” method. After principal component analysis (PCA), the 24 most powerful PCs were used for t-distributed stochastic neighbor embedding (t-SNE) analysis for dimension reduction. Subsequently, cells were divided into fourteen clusters with a resolution of 0.5 by KNN analysis and the “FindClusters” method, and cell types were subsequently annotated by specific cell markers as previously described^30–32^. Finally, cell–cell communications among the cell types were investigated by the “CellChat” package^33^.

### Differential analysis between the two groups separated by GRS

In the training cohort, patients were divided into two groups according to the optimal cutoff value of the GRS. Differentially expressed genes (DEGs) between the two groups were identified by the “DESeq2” package according to the count data, while the functional differences between the two groups were revealed by gene set enrichment analysis (GSEA). Next, functional enrichment analyses, including Gene Ontology (GO) and Kyoto Encyclopedia of Genes and Genomes (KEGG)^34,35^, were performed on the significant DEGs (P < 0.05 and |log2FoldChange| > 1). All of the functional analyses in this study were conducted by the “clusterProfiler” package^36^.

## Results

### Construction and efficiency evaluation of MRS

**Figure 1:**
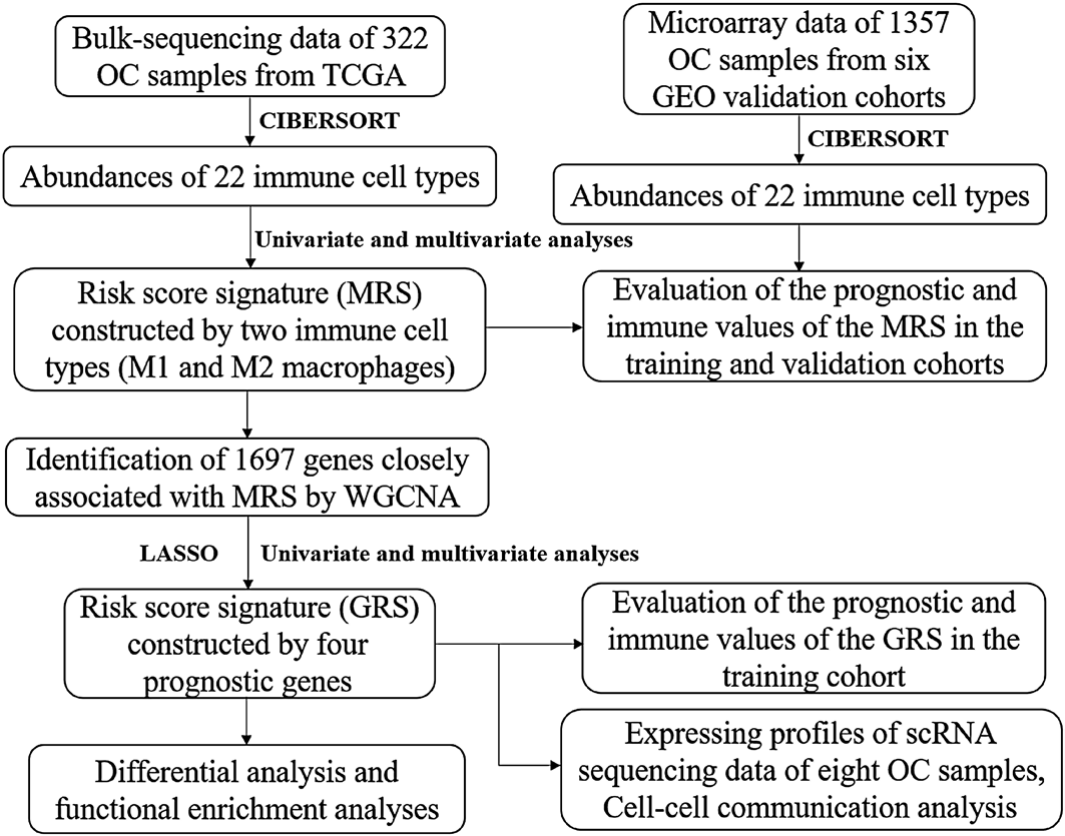
Flowchart of the study.

**Figure 2:**
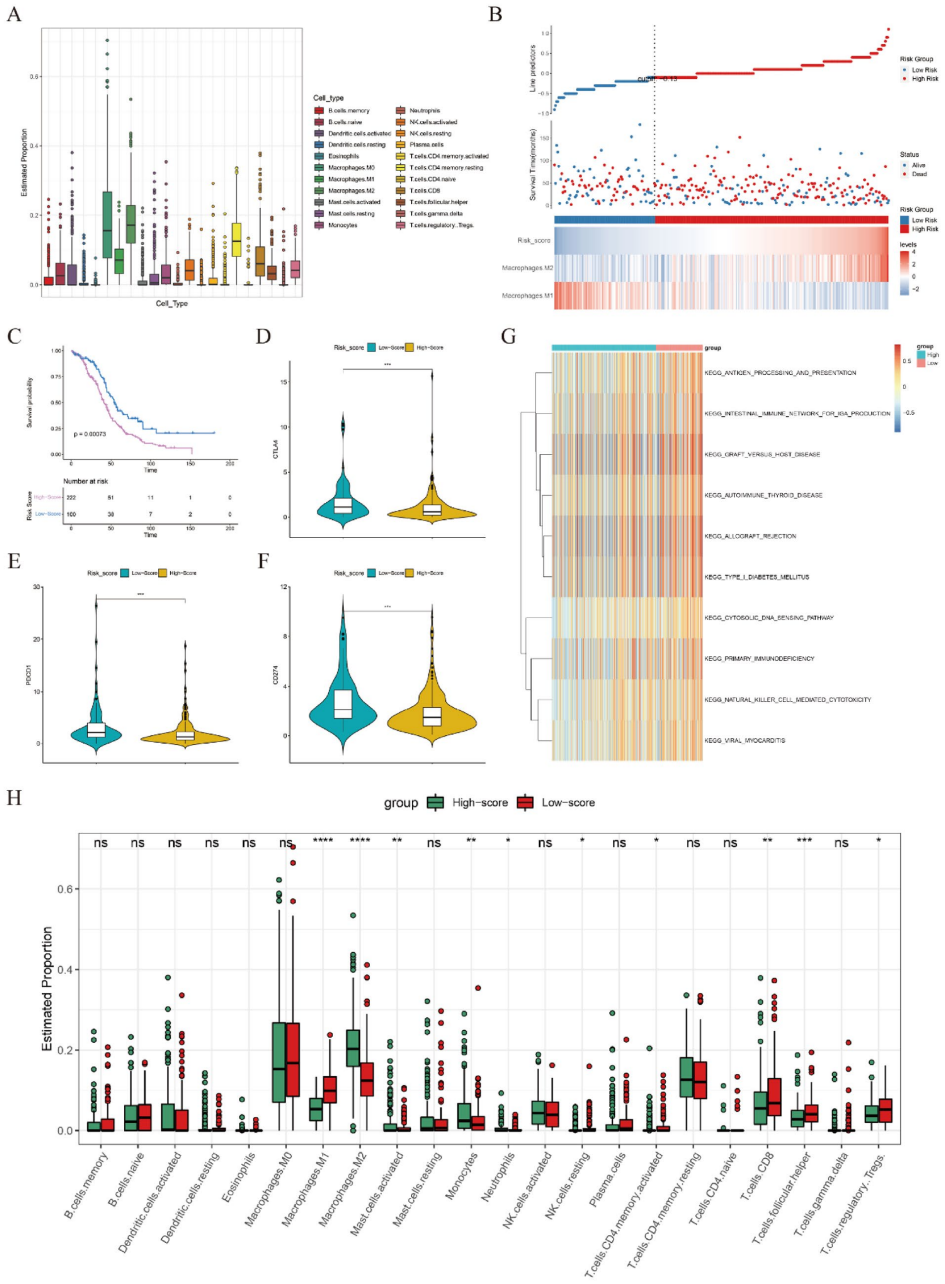
Immune and survival analyses in the training cohort. (**A**) The overall abundances of 22 immune cell types across 322 OC samples. (**B**) Cutoff value (upper panel), survival status (middle panel) and expression heatmap of M1 and M2 macrophages (lower panel). (**C**) Kaplan–Meier plotter curve between the high- and low-score groups separated by the optimal cut-off value of MRS. (**D**–**F**) The expression levels of CTLA-4, PDCD1 and CD274 between the high- and low-score groups. (**G**) GSVA between the two risk groups. (**H**) The expression levels of 22 immune cell types between the high- and low-score groups. *p < 0.05, **p < 0.01, ***p < 0.001. *ns* not significant.

The flowchart of this study is shown in Fig. 1. The overall abundances of 22 immune cell types across the 322 OC samples in the training cohort were calculated by CIBERSORT (Fig. 2A). We noticed that the most enriched immune cell types in OC were M0 macrophages, M1 macrophages, M2 macrophages, resting memory CD4 T cells and CD8 T cells. Next, significant prognostic immune cells (M1 macrophages and M2 macrophages, P < 0.05) screened by univariate and multivariate Cox analyses (Table S1) were used to construct the MRS according to the following formula: abundances of M1 macrophages × (− 4.056590) + abundances of M2 macrophages × (2.323889). OC patients were separated into high- and low-score groups according to the optimal cutoff value automatically calculated by the “roc” method in the “ggrisk” package; the group information, survival status and abundances of M1 macrophages and M2 macrophages between the two groups are visualized in Fig. 2B. The Kaplan–Meier plotter curve showed that patients in the low-score group had a significantly better prognosis than those in the high-score group (Fig. 2C). To further clarify the mechanisms of MRS affecting prognosis, differences in the immune landscape between the two groups were systematically investigated. We found that the expression levels of the major immune checkpoints (CTLA-4, PDCD-1 and CD274) were both significantly highly expressed in the low-score group (Fig. 2D–F). In addition, as revealed by GSVA, some immune-related terms were significantly enriched in the low-score group, such as antigen processing and presentation, intestinal immune network for IgA production and NK cell-mediated cytotoxicity (Fig. 2G). Furthermore, the results showed that M1 macrophages, resting NK cells, activated memory CD4 T cells, CD8 T cells, follicular helper T cells and regulatory T cells were significantly enriched in the low-score group, while M2 macrophages, activated mast cells, monocytes and neutrophils were significantly enriched in the high-score group (Fig. 2H). Next, the correlation between clinicopathological features and risk score was explored. As shown in Fig. S1A, patients in the “≥ 60 years” group had a higher MRS than the one in the “< 60 years” group, while there was no significant difference between groups with other clinicopathological features (Fig. S1B–F). Taken together, these results indicated that MRS showed good performance in prognosis prediction, that it could divide OC patients into two significantly different immune cell landscapes, and that patients in the low-score group (immune subtype) may have a better therapeutic response to ICIs.

### Efficiency validation of the MRS

**Figure 3:**
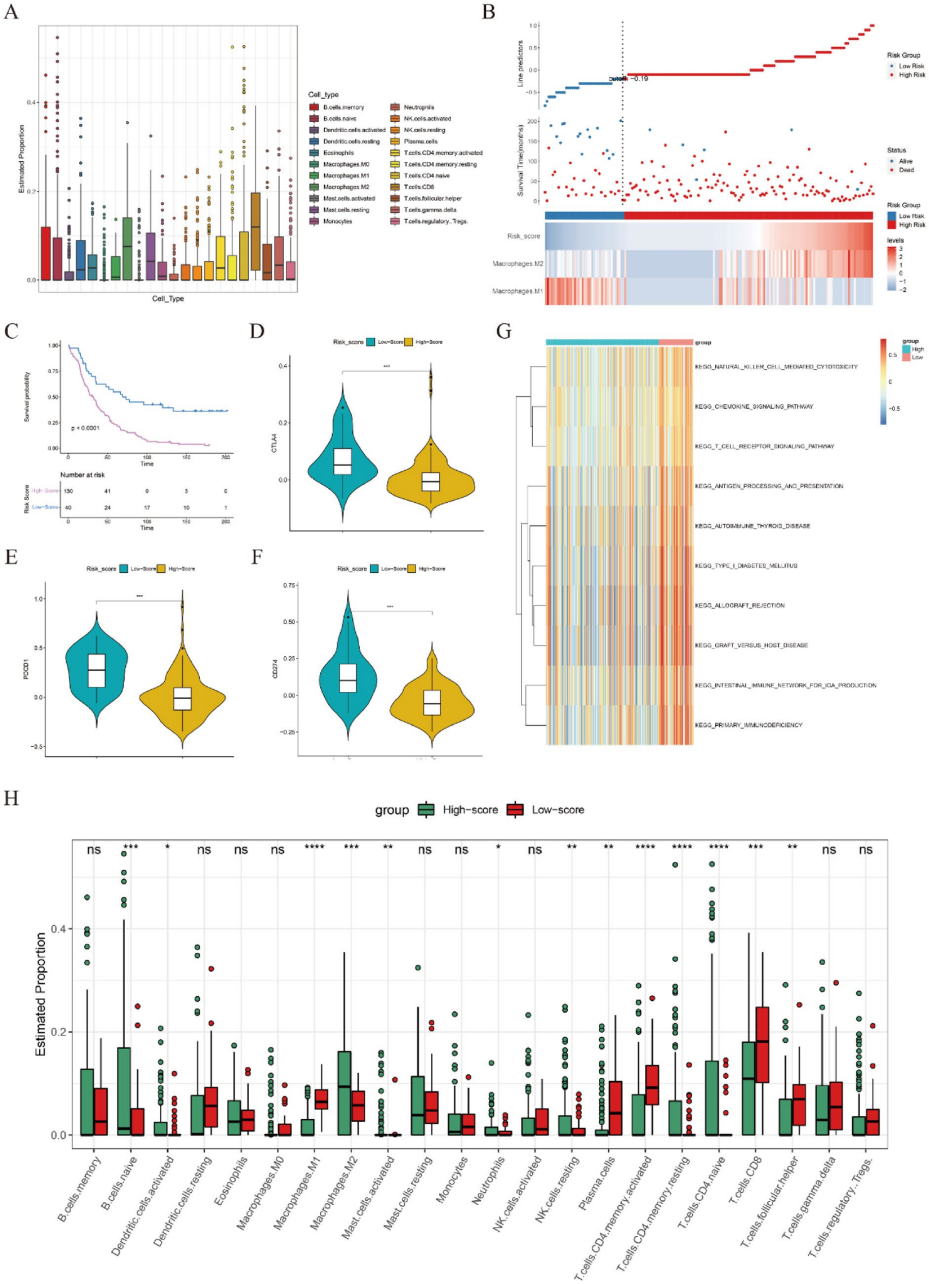
Immune and survival analyses in the GSE53963 dataset. (**A**) The overall abundances of 22 immune cell types across 170 OC samples. (**B**) Cutoff value (upper panel), survival status (middle panel) and expression heatmap of M1 and M2 macrophages (lower panel). (**C**) Kaplan–Meier plotter curve between the high- and low-score groups separated by the optimal cutoff value of MRS. (**D**–**F**) The expression levels of CTLA-4, PDCD1 and CD274 between the high- and low-score groups. (**G**) GSVA between the two risk groups. (**H**) The expression levels of 22 immune cell types between the high- and low-score groups. *p < 0.05, **p < 0.01, ***p < 0.001. *ns* not significant.

To further validate the efficiency of the MRS, a total of 170 OC patients in GSE53963 were comprehensively analyzed. First, the abundances of 22 immune cells were estimated by CIBERSORT (Fig. 3A). Next, the MRS was calculated by the same formula used for the training cohort, and OC patients were divided into two groups according to the optimal cutoff value automatically calculated by the “roc” method in the “ggrisk” package. The results of survival analyses showed that patients in the low-score group had a significantly better prognosis than those in the high-score group, consistent with the results in the training cohort (Fig. 3B,C). Moreover, the prognostic values of the MRS were further validated in the other five GEO datasets (Fig. S2A–E). The details of these GEO datasets are listed in Table S2. Furthermore, significantly high expression of immune checkpoints (CTLA-4, PDCD-1 and CD274) was observed in the low-score group in the GSE53963 dataset (Fig. 3D–F). Some immune-related terms were significantly enriched in the low-score group, such as NK cell-mediated cytotoxicity, antigen processing and presentation and the T cell receptor signaling pathway, as revealed by GSVA (Fig. 3G). Finally, the expression levels of immune cells between the two groups were explored. As shown in Fig. 3H, M1 macrophages, plasma cells, activated memory CD4 T cells, CD8 T cells and follicular helper T cells were significantly enriched in the low-score group, while naïve B cells, activated dendritic cells, M2 macrophages, activated mast cells, neutrophils, resting NK cells, resting memory CD4 T cells and naïve CD4 T cells were significantly enriched in the high-score group. These results indicated that MRS showed excellent performance in prognosis prediction and immune landscape discrimination in the validation cohorts.

### Identification of MRS related hub genes

**Figure 4:**
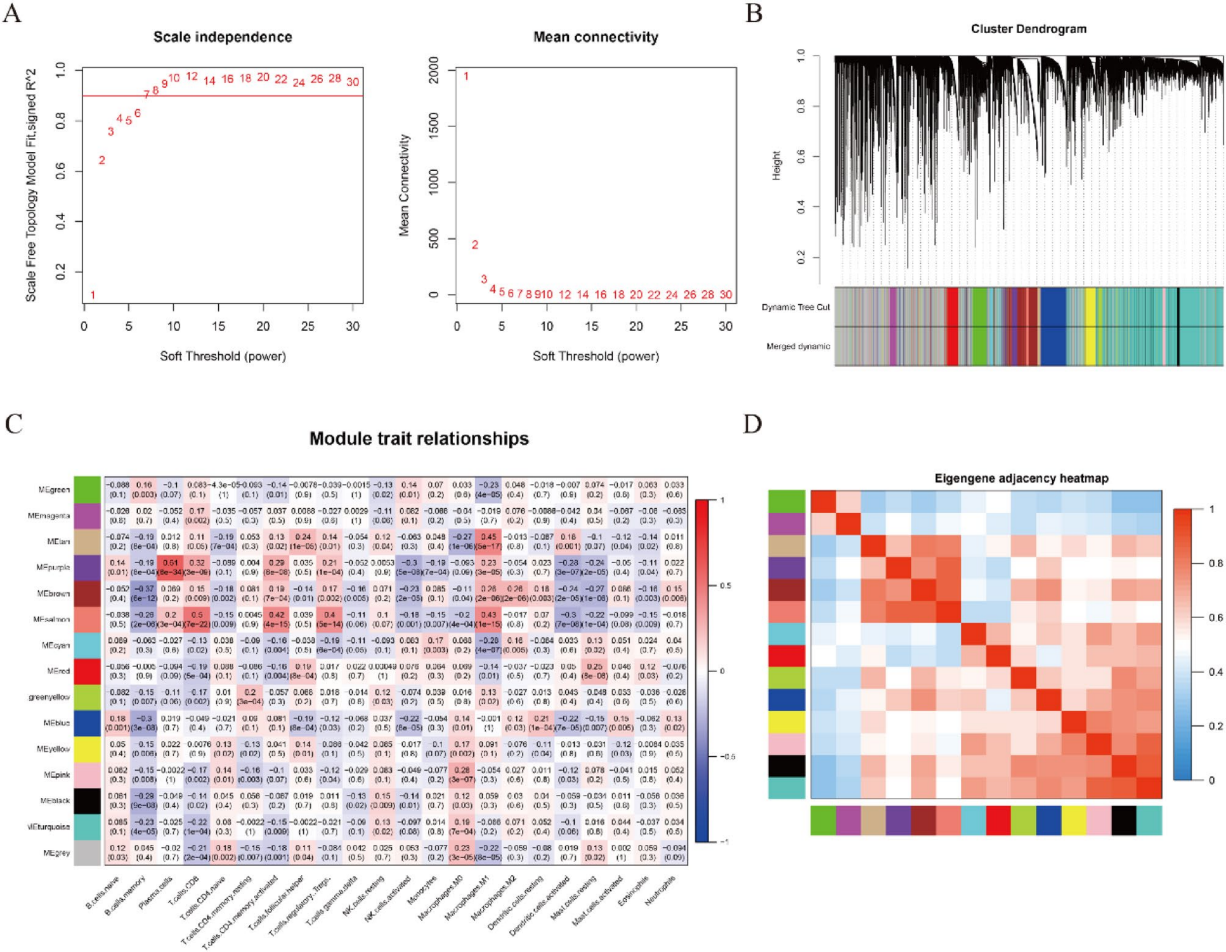
Identification of MRS-related gene modules. (**A**) Diagrams of scale independence and mean connectivity for the identification of soft threshold power. (**B**) Sample cluster dendrogram of OC patients in the training cohort. (**C**) Correlation heatmap between modules and immune cell types. (**D**) Correlation heatmap between modules.

To identify the hub genes that are closely associated with M1 and M2 macrophages, WGCNA was conducted in the training cohort. According to the soft threshold power = 7, genes were divided into fifteen coexpression modules (Fig. 4A,B). In general, purple and salmon modules showed a stronger correlation with more immune cells, including M1 macrophages and CD8 T cells (Fig. 4C). From another perspective, M1 macrophages were closely associated with the tan, purple, brown and salmon modules, while M2 macrophages were closely associated with the brown module (Fig. 4C). Considering that these four modules (tan, purple, brown and salmon) were also closely associated with each other (Fig. 4D), a total of 1697 genes in these modules were selected for further analyses.

### Construction and evaluation of GRS

**Figure 5:**
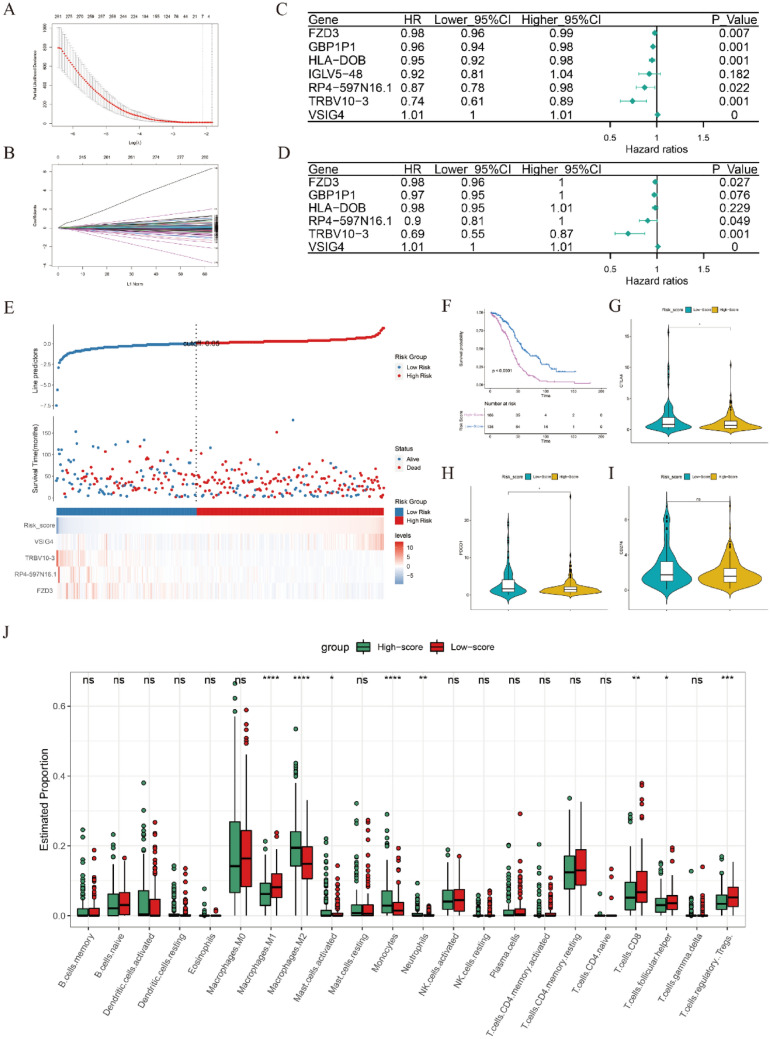
Construction an evaluation of the GRS. (**A**,**B**) Cvfit and fit plots of LASSO screen. (**C**) Forest plot of univariate Cox analysis. (**D**) Forest plot of multivariate Cox analysis. (**E**) Cutoff value (upper panel), survival status (middle panel) and expression heatmap of the four significant prognostic genes (FZD3, RP4-597N16.1, TRBV10-3 and VSIG4) (lower panel). (**F**) Kaplan–Meier plotter curve between the high- and low-score groups separated by the optimal cutoff value of GRS. (**G**–**I**) The expression levels of CTLA-4, PDCD1 and CD274 between the high- and low-score groups. (**J**) The expression levels of 22 immune cell types between the high- and low-score groups. *p < 0.05, **p < 0.01, ***p < 0.001, ns: not significant.

To explore whether these genes have MRS-related functions, survival and immune-related analyses were performed. The seven prognostic hub genes screened by LASSO (Fig. 5A,B) were further analyzed by univariate and multivariate Cox analyses (Fig. 5C,D). Subsequently, a risk score (GRS) was constructed based on remaining four most significant prognostic genes (FZD3, RP4-597N16.1, TRBV10-3 and VSIG4; P < 0.05) and their corresponding risk coefficients as follow: expression levels of FZD3 × (− 0.021642228) + expression levels of RP4-597N16.1 × (− 0.108795364) + expression levels of TRBV10-3 × (− 0.498230068) + expression levels of VSIG4 × (0.008368181). OC patients were separated into two groups according to the optimal cutoff value of GRS calculated by the “roc” method; the group information, survival status and expression levels of the abundances of these four hub genes of the two groups are visualized in Fig. 5E. The Kaplan–Meier plotter curve showed that patients in the low-score group had a significantly better prognosis than those in the high-score group (Fig. 5F). Consistent with the MRS results, the expression levels of the immune checkpoints (CTLA-4, PDCD-1 and CD274) were also significantly highly expressed in the low-score group (Fig. 5G–I). Moreover, the results of immune analyses showed that M1 macrophages, CD8 T cells, follicular helper T cells and regulatory T cells were significantly enriched in the low-score group, while M2 macrophages, activated mast cells, monocytes and neutrophils were significantly enriched in the high-score group (Fig. 5J). Next, the correlation between clinicopathological features and risk score was explored. As shown in Fig. S1G,H, patients in the “stage III–IV” group and in the “nonwhite” group had a higher GRS than those in the “stage I–II” group and the “white” group, while there was no significant difference in GRS between groups with other clinicopathological features (Fig. S1I–L). It is noteworthy that the most effective gene for GRS construction is TRBV10-3 (|risk efficiency| = 0.498230068), which is a T cell receptor. These results indicated that the GRS also exhibited excellent performance in prognosis prediction and immune cell landscape discrimination, similar to the results of MRS, and M1 and M2 macrophages may affect the prognosis of OC patients by regulating T cell function.

### Processing of scRNA sequencing data and cell–cell communication analysis

**Figure 6:**
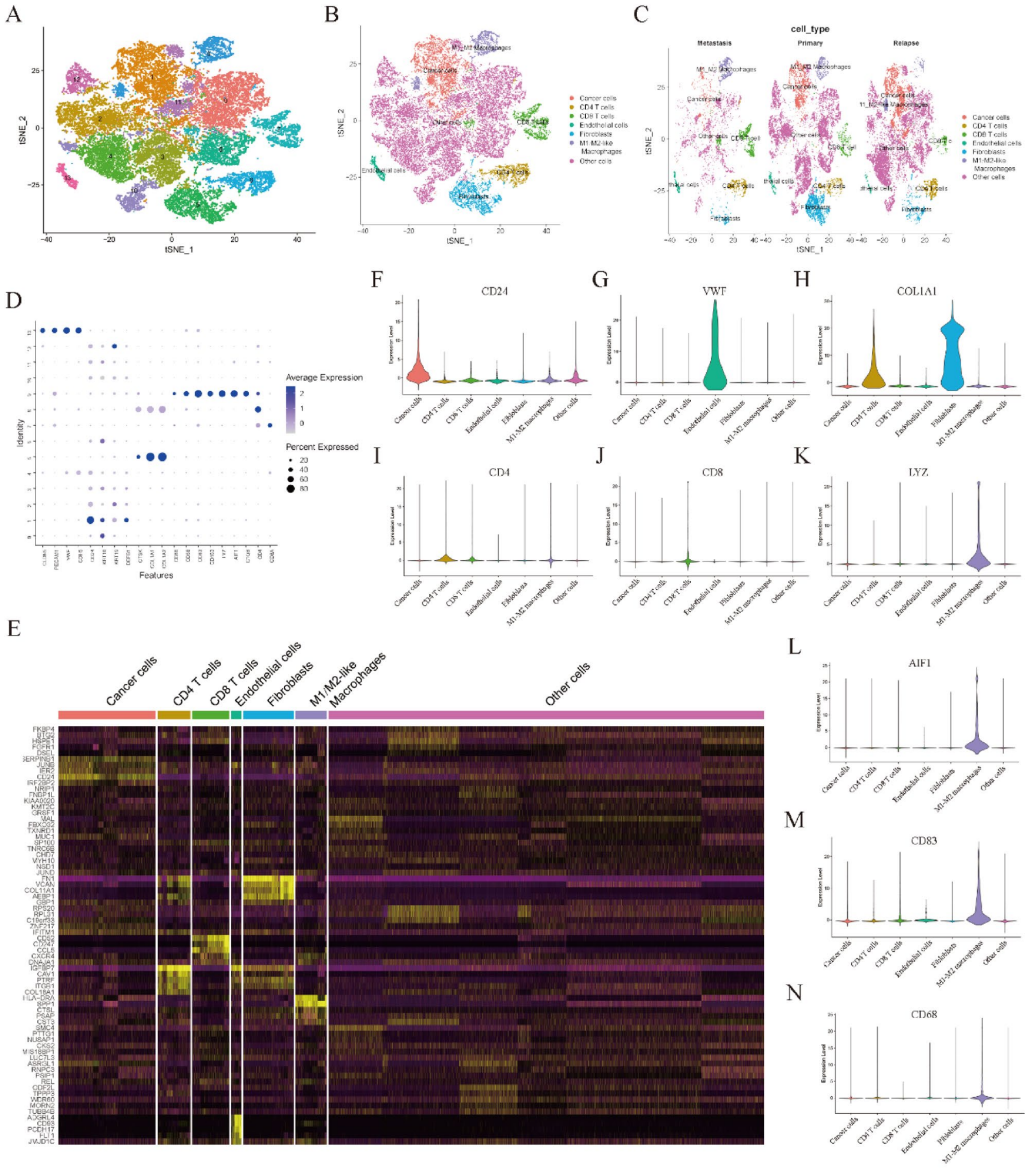
The results of scRNA sequencing data analysis. (**A**) t-SNE plot of 32,078 cells from eight OC samples. (**B**,**C**) t-SNE plot showing the annotations for seven cell types. (**D**) Expression profiles of the marker genes in each cell type. (**E**) Heatmap of significant DEGs in each cell type. (**F**–**N**) Specific marker genes of cancer cells (**F**), endothelial cells (**G**), fibroblasts (**H**), CD4 T cells (**I**), CD8 T cells (**J**), myeloid-derived cells (**K**, **L**), M1-like macrophages (**M**) and M2-like macrophages (**N**).

**Figure 7:**
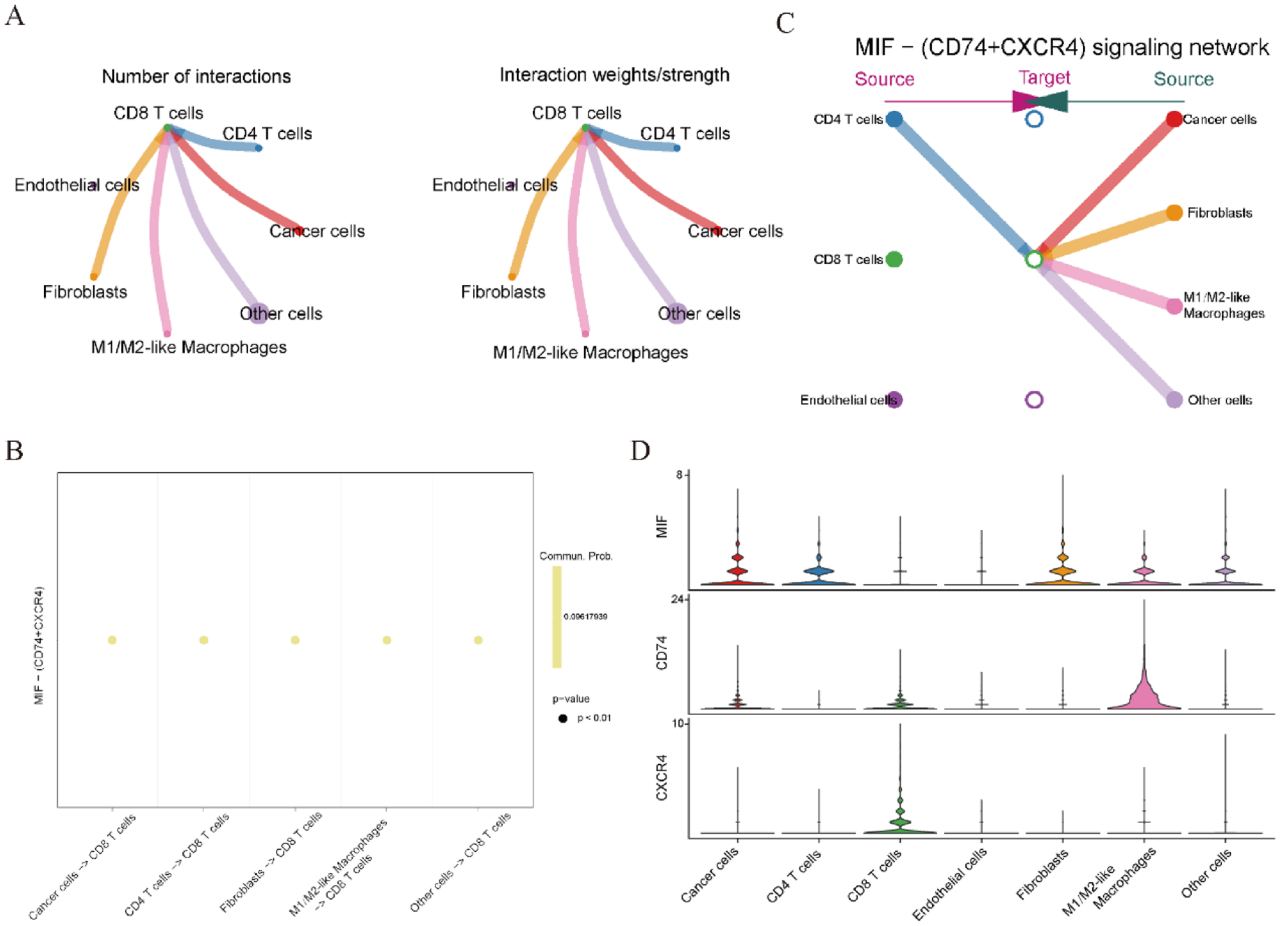
The results of cell–cell communication analysis revealed by scRNA sequencing data. (**A**) Number (left panel) or strength (right panel) of the interactions among the immune cells. (**B**,**C**) The possible incoming or outgoing signaling pathways among the immune cells. (**D**) The expression levels of MIF-CD74/CXCR4 signaling molecules among these cell types were visualized by violin plot.

To further elucidate the potential interactions between macrophages and T cells, scRNA sequencing data were analyzed. After data filtering, a total of 32,078 cells from eight samples remained for further analysis. The expression profiles of primary, metastatic and relapsed tumor cells, as well as the correlations between nFeature-RNA and nCount-RNA, are visualized in Figure S3A,B. Next, a total of fourteen clusters were identified across all of the cancer cells, and they were annotated to seven cell types (cancer cells, CD4 T cells, CD8 T cells, endothelial cells, fibroblasts, M1/M2-like macrophages and other cells) according to the specific markers as previously described (Fig. 6A–D). Specific markers for cell annotation are listed in Table S3. As shown in Fig. S3C, there were no significant batch effects caused by the cell cycle. The significant DEGs in each cell type are visualized in Fig. 6E. Violin plots and feature plots were used to visualize the marker genes in each cell type (Fig. 6F–N and Fig. S3D–L). Next, cell–cell communication analysis was conducted across these cell types. The results showed that CD8 T cells mainly acted as signal receivers which could receive signals from the other five cell types except endothelial cells (Fig. 7A). Next, the potential signaling pathways between these cell types were investigated by internal secreted signaling in the “CellChat” package. The results indicated that the potential signaling pathway between CD8 T cells and other cell types was MIF-CD74/CXCR4 (Fig. 7B,C). A violin plot was subsequently used to visualize the expression levels of MIF-CD74/CXCR4 signaling molecules in these cell types (Fig. 7D). Interestingly, the outgoing molecule CD74 was significantly highly expressed in M1/M2-like macrophages, while the ingoing molecule CXCR4 was significantly highly expressed in CD8 T cells. These results suggested that cell–cell communication between M1/M2-like macrophages and CD8 T cells may represent the most important pairs in OC. Taken together, these results indicated that M1/M2-like macrophages may affect the prognosis of OC patients by regulating CD8 T cells function through cell–cell communication.

### Differential analysis

**Figure 8:**
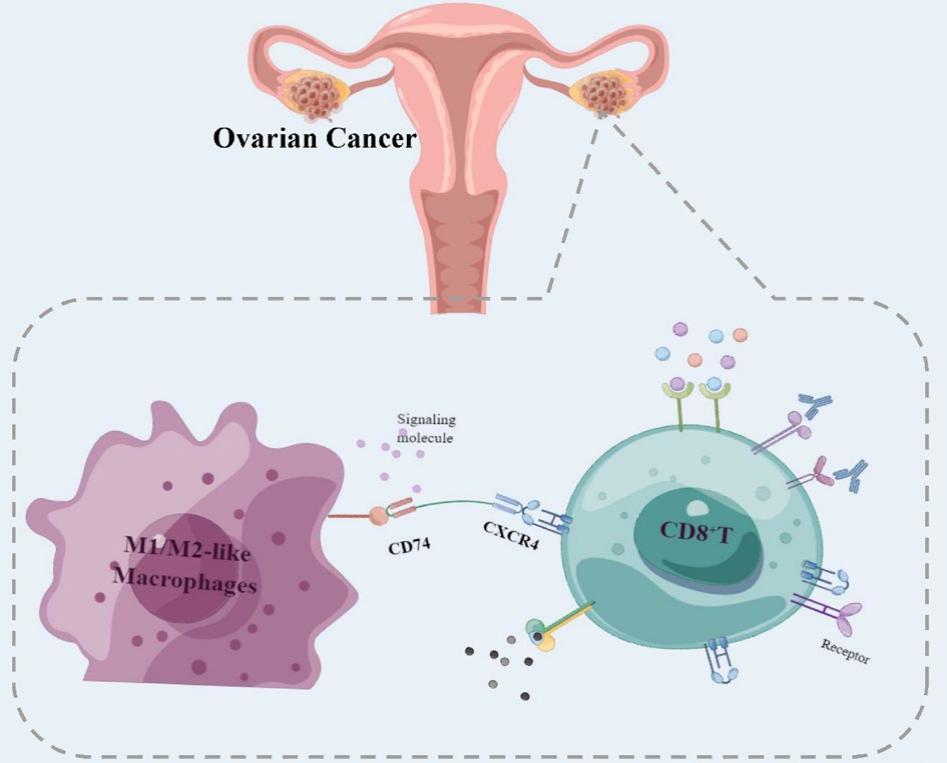
Hypothetic scheme.

To systematically clarify the differences between the two groups separated by GRS, differential analysis and functional enrichment analyses were conducted in the training cohort. The significant DEGs between the two groups are shown in Fig. S4A,B. We noticed that TRBV10-3 and RP4-597N16.1 were both in the top five significantly highly expressed genes in the low-score group (Fig. S4B). The GSEA results revealed that there were significant differences in some immune-related terms between the two groups, such as lymphocyte-mediated immunity, regulation of the immune effector process, negative regulation of the immune system process, activation of the innate immune response and B cell-mediated immunity (Fig. S4C–G). Next, GO and KEGG analyses were conducted based on the significant DEGs. As shown in Fig. S4H, the most significant results of GO analyses were also immune-related terms such as humoral immune response and complement activation in biological process (BP), immunoglobulin complex and T cell receptor complex in cellular components (CC), and antigen binding and immunoglobulin receptor binding in molecular function (MF). The most significant terms in KEGG analysis were both associated with signaling transfer such as cytokine-cytokine receptor interactions and chemokine signaling pathways (Fig. S4I). These results further clarified the differences in immune-related terms and immune-related signaling pathways between the groups of OC patients separated by GRS. Hypothetic scheme of research results was visualized in Fig. 8.

## Discussion

OC is a life-threatening gynecological cancer with limited therapeutic options^1,2^. Immunotherapy using ICIs and CAR-T cells has shown excellent efficacy in a variety of tumors, but the response of OC to immunotherapy is still unsatisfactory^15,18–21^. Therefore, it is urgent to clarify the immune characteristics of OC to construct a model for immunotherapy response prediction and to search for synergetic therapeutic targets.

In this study, immune cell abundances in OC patients were calculated by CIBERSORT, and a risk score (MRS) based on the most significant prognostic immune cell types (M1 and M2 macrophages) were subsequently constructed. According to the risk coefficients, M1 macrophages (also known as classically activated macrophages) were identified as a protective factor in OC, while M2 macrophages (also known as alternatively activated macrophages) were identified as an adverse factor in OC, consistent with previous research^37–39^.

Macrophage polarization has been reported to play an important role in a variety of tumor pathological processes, such as carcinogenesis and metastasis, and in chemotherapy and immunotherapy responses^40–43^. Promoting the polarization of M2 macrophages to M1 macrophages is an important method for antitumor therapy^38,44,45^. However, the integrated role of M1 and M2 macrophages in the prediction of prognosis and response to immunotherapy in OC patients remains largely unknown. In this study, we constructed a prognostic risk score (MRS) based on the abundances and risk coefficients of M1 and M2 macrophages in OC for the first time. It is noteworthy that this risk score signature can well distinguish OC patients into two immune characteristic landscapes. These results suggested that MRS can effectively identify the immune subtypes of OC patients who have a better prognosis and may have a sensitive response to ICIs.

It has been reported that macrophages can affect the immune response in many ways, such as releasing self-activatable photo-extracellular vesicles^46^, delivering exosomes carrying functional molecules such as microRNA and delivering drugs as messengers^47,48^. However, the specific macrophage-related molecules that may mediate the immune function in OC remain unknown. In this study, WGCNA was conducted to identify the hub genes that were closely associated with M1 and M2 macrophages, which may mediate the functions of macrophages in OC. After subsequent survival analyses, a macrophage-related gene risk score (GRS) was constructed based on the four significant prognostic hub genes (FZD3, RP4-597N16.1, TRBV10-3 and VSIG4). Consistent with the MRS results, the immune subtype with better prognosis and an inflammatory immune microenvironment can also be identified by GRS. These results indicated that targeting the hub genes for the construction of GRS may enhance the immune response of OC to immunotherapy. It is noteworthy that the most effective gene (TRBV10-3) for the construction of GRS is a T cell receptor, which indicated that macrophages may play a role in OC by affecting T cell function.

The function of T cells can be regulated by many immune microenvironment factors, such as M1 macrophage repolarization, B cell paracrine secretion and cross-talk among different T cell subsets^49–51^. Recently, relevant research results from chimeric antigen receptor macrophages (CAR-Ms) have shown that they can induce a pro-inflammatory tumor microenvironment and boost anti-tumor T cell activity^52,53^. To further investigate the potential signaling communication between macrophages and T cells in OC, cell–cell communication analysis was conducted by scRNA sequencing data. As revealed in Fig. 7, the most important signaling pair among different cell types in OC was MIF-CD74/CXCR4 between M1/M2-like macrophages and CD8 T cells. This result further emphasized the central role of the signaling communications between M1/M2-like macrophages and CD8 T cells, which may be potential synergetic therapeutic targets for ICI treatment in OC patients.

However, there are still some limitations to this study. First, the values of the GRS for prognosis prediction and immune landscape discrimination need to be validated in more real-world OC patient cohorts because some of the four prognostic genes could not be detected in the major microarray platforms. Second, whether patients with the immune subtype have a better response to ICIs needs to be further studied in clinical trials. Finally, whether the hub genes used for the construction of the GRS can become synergetic therapeutic targets of ICIs in OC needs to be further verified by in vitro and in vivo experiments.

In summary, two risk score models were constructed based on M1 and M2 macrophages and their functionally related hub genes. The low-score groups of these two models were identified as immune subtypes, patients with a better prognosis and an inflammatory immune microenvironment. The signaling pathway molecules between M1/M2-like macrophages and CD8 T cell communication may be potential synergetic therapeutic targets of ICI treatment in OC patients.

## Supplementary Information

Supplementary Information.

## Data Availability

The datasets presented in this study can be found in online repositories. In detail, transcriptome data of OC from TCGA were downloaded from UCSC-Xena (https://xena.ucsc.edu/), while the clinicopathological data were downloaded from cBioPortal (https://www.cbioportal.org/). Microarray data of OC patients were downloaded from the GEO database (https://www.ncbi.nlm.nih.gov/geo/).
