# Supplementary material for: Identification of the immune subtype of ovarian cancer patients by integrated analyses of transcriptome and single-cell sequencing data
Source: Sci Rep. 2022 Aug 2;12:13296. doi: 10.1038/s41598-022-17645-7 (PMC9346122; doi:10.1038/s41598-022-17645-7)
Supplement: Supplementary file 1 — Supplementary Information. [file 41598_2022_17645_MOESM1_ESM.pdf]

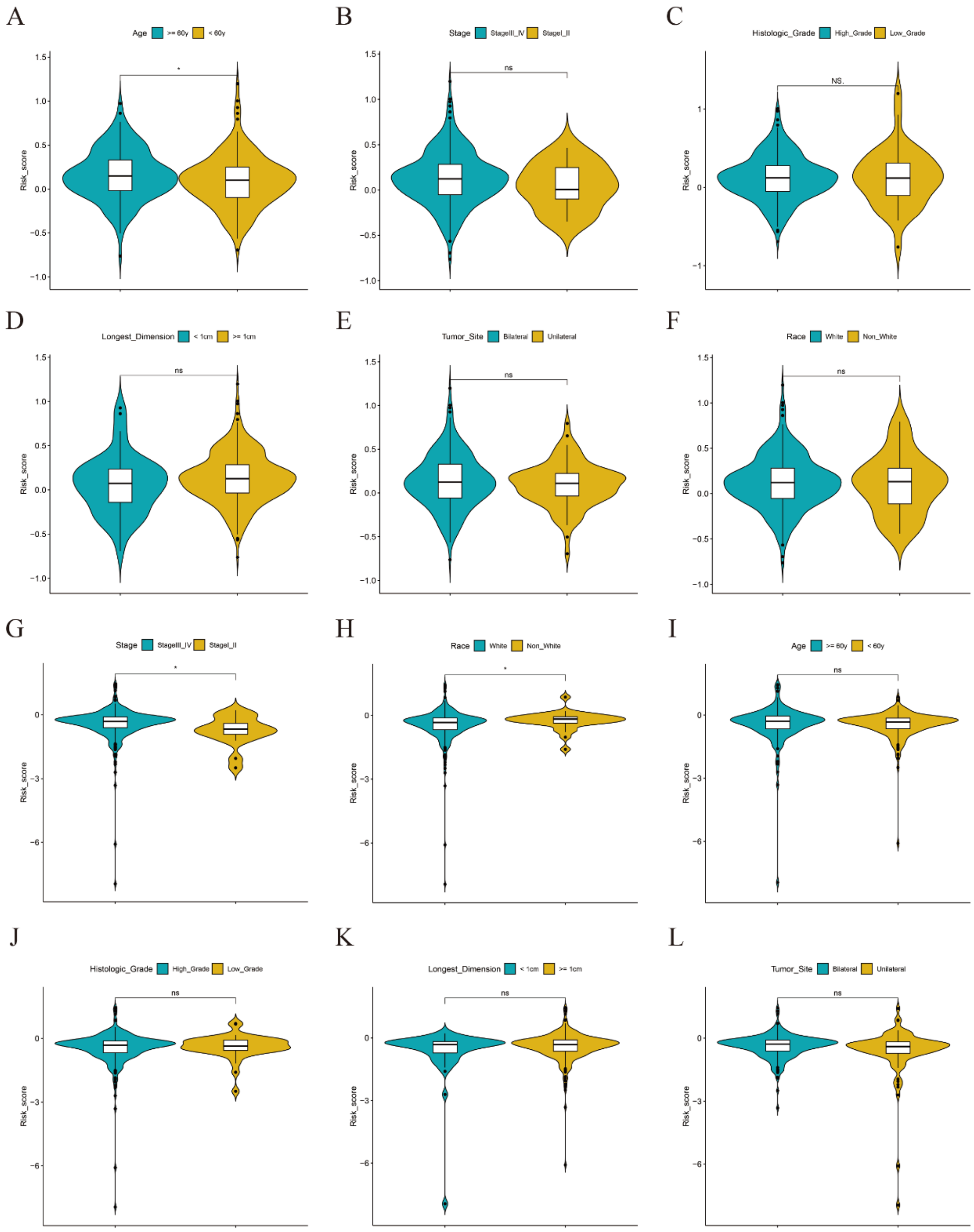

Figure S1. Correlations between different clinicopathological features and risk scores. Relative expression levels of MRS between different age (A), stage (B), histological grade (C), longest dimension (D), tumor site (E) and race (F) groups. Relative expression levels of GRS between different stage (G), race (H), age (I), histological grade (J), longest dimension (K) and tumor site (L) groups. *p < 0.05, **p < 0.01, ***p < 0.001, ns: not significant.

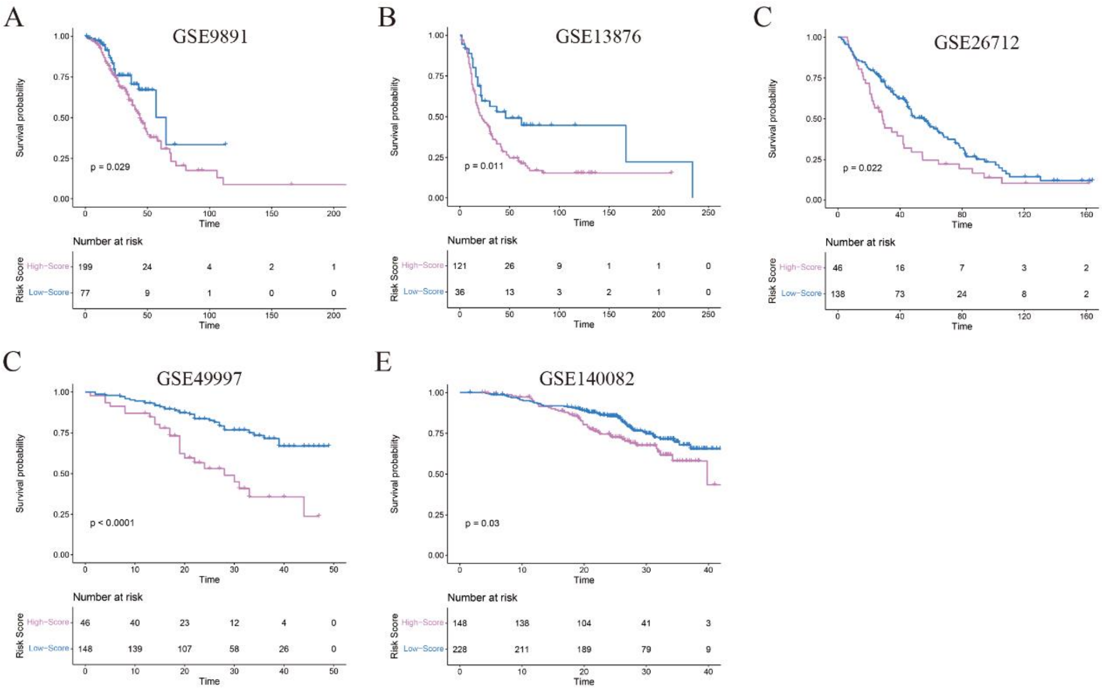

Figure S2. External validation of the prognostic value of the MRS. Kaplan-Meier plotter curve of the GSE9891 (A), GSE13876 (B), GSE26712 (C), GSE49997 (D) and GSE140082 (E).

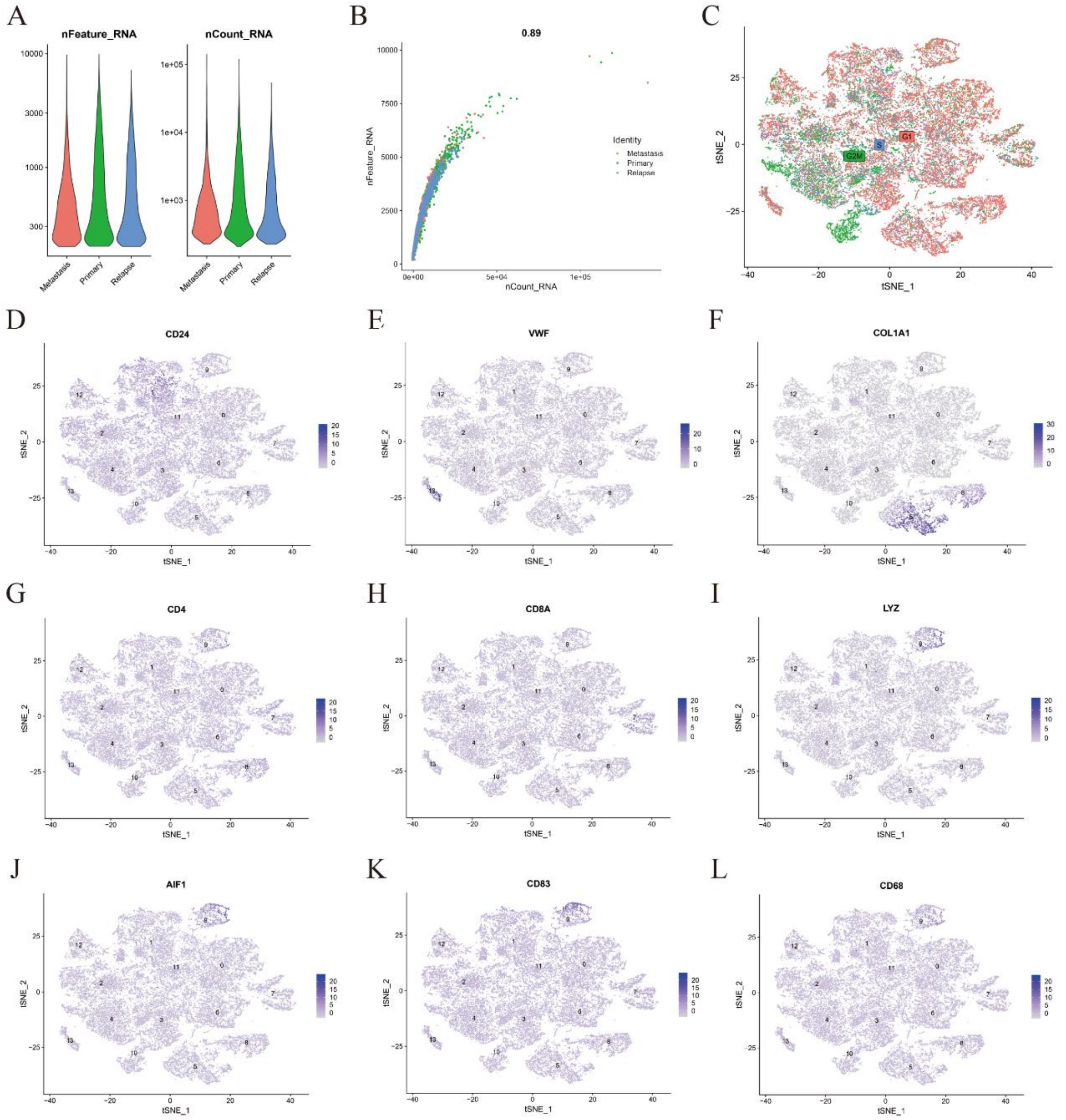

Figure S3. Quality control and cell annotation of the OC scRNA data. (A) Expression profiles of primary, metastasis and relapse tumor cells. (B) Correlations between nFeature-RNA and nCount-RNA. (C) Cell cycle distribution after dimensionality reduction. (d-l) Specific marker genes of the cancer cells (D), endothelial cells (E), fibroblasts (F), CD4 T cells (G), CD8 T cells (H), myeloid-derived cells (I, J), M1 macrophages (K) and M2 macrophages (L).

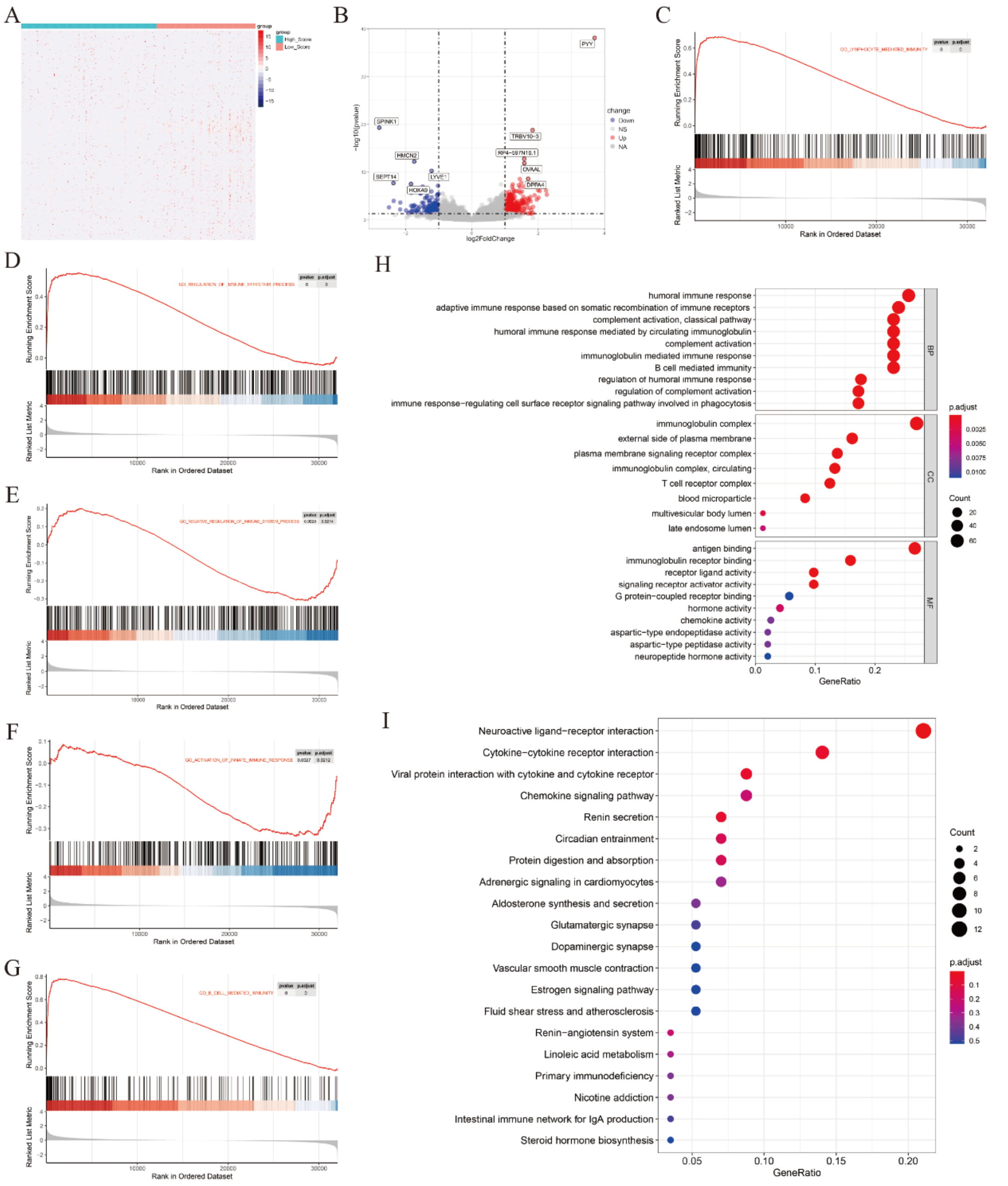

Figure S4. Differential analysis and functional enrichment analyses between the two groups separated by GRS in the training cohort. (A) Heatmap of the DEGs. (B) Volcano plot of the DEGs. (C-G) Representative immune-related results of GSEA. (H) Dot plot of the results of GO analysis. (I) Dot plot of the results of KEGG analysis.

	Univariate analysis		Multivariate analysis	
Cell types	HR (95% CI)	P	HR (95% CI)	P
Memory B cells	0.78 (0.01-42.51)	0.904	NA	NA
Naïve B cells	2.2 (0.11-45.19)	0.61	NA	NA
Activated Dendritic cells	0.8 (0.1-6.34)	0.834	NA	NA
Resting Dendritic cells	179.07 (0.58-55329.23)	0.076	NA	NA
Eosinophils	5.68 (0-984467329385.04)	0.895	NA	NA
M0 Macrophages	0.87 (0.31-2.45)	0.793	NA	NA
M1 Macrophages	0.02 (0-0.26)	0.004	0.03 (0-0.5)	0.015*
M2 Macrophages	12.82 (2.38-69.16)	0.003	8.78 (1.69-45.76)	0.01*
Activated Mast cells	185.08 (2.93-11673.84)	0.014	24.12 (0.34-1720.34)	0.144
Resting Mast cells	0.24 (0.01-4.89)	0.356	NA	NA
Monocytes	15.96 (0.89-287.19)	0.06	NA	NA
Neutrophils	165741.16(0.41-6.68E+10)	0.068	NA	NA
Activated NK cells	3.92 (0.12-124.07)	0.439	NA	NA
Resting NK cells	0.2 (0-13072.46)	0.775	NA	NA
Plasma cells	0.01 (0-1.5)	0.073	NA	NA
Activated memory CD4 T cells	0 (0-7.36)	0.153	NA	NA
Resting memory CD4 T cells	0.52 (0.07-3.71)	0.518	NA	NA
Naive CD4 T cells	0.26 (0-426636.77)	0.855	NA	NA
CD8 T cells	0.83 (0.09-7.3)	0.863	NA	NA
Follicular helper T cells	0.11 (0-9.39)	0.33	NA	NA
Gamma delta T cells	0.06 (0-827.74)	0.568	NA	NA
Regulatory T cells	0.14 (0-8.37)	0.348	NA	NA

*$p < 0.05$.

Table S1. Univariate and multivariate Cox regression analysis of the 22 immune cell types.

GEO accession	Patient number	Platform	Country
GSE53963	170	GPL6480	USA
GSE9891	276	GPL570	Australia
GSE13876	157	GPL7759	Netherlands
GSE26712	184	GPL96	USA
GSE49997	194	GPL2986	Austria
GSE140082	376	GPL14951	USA

Table S2. Detail information of the GEO datasets analyzed in this study.

Cell Type	Cell Marker
T cells	CD3D, CD3E, CD3G, CD4, CD8A, CD8B
B cells	MS4A1, CD79A, CD79B
Epithelial cancer cells	EPCAM, PAX8, CD24, CDH1, KRT18, KRT19
Ovarian stroma cells	FOXL2
Myeloid cells	CD68, LYZ, AIF1, C1QB
Fibroblasts	COL1A1, COL1A2
Endothelial cells	CLDN5, PECAM1, VWF, CDH5
NK cells	KLRF1, KLRD1
Epithelial cells	EPCAM, KRT19, DEFB1, CTSK
M2 Macrophages	CD169, CX3CR1, CD83, CD163, CD206
M1 Macrophages	CD80, CD86, CD68

Table S3. Specific markers for cell annotation.
